# Strategies to Improve Women’s Leadership Preparation for Early Career Global Health Professionals: Suggestions from Two Working Groups

**DOI:** 10.5334/aogh.3705

**Published:** 2022-07-11

**Authors:** Meagan Harrison, Dan N. Tran, Andressa Pena, Sloka Iyengar, Aisha Ahmed Abubakar, Katarina Hoernke, Yetunde O. John-Akinola, Sandra Kiplagat, Agustina M. Marconi, Tanaz M. Vaghaiwalla, Anna Kalbarczyk, Jennifer L. Weinberg

**Affiliations:** 1 Department of International Health, Johns Hopkins Bloomberg School of Public Health, Baltimore, MD, USA; 2 Johns Hopkins Center for Global Health, Johns Hopkins University, Baltimore, MD, USA; 3 Department of Pharmacy Practice, Temple University School of Pharmacy, Philadelphia, PA, USA; 4 Infectious Disease & Microbiology-Public Health Department, University of Pittsburgh, Pittsburgh, PA, USA; 5 American Museum of Natural History, New York, NY, USA; 6 Department of Community Medicine, Ahmadu Bello University Zaria, Kaduna, Nigeria; 7 Institute for Global Health, University College London, London, UK; 8 Department of Health Promotion and Education, University of Ibadan College of Medicine, Ibadan, Nigeria; 9 Department of Epidemiology, Florida International University, Miami, FL, USA; 10 University Health Services, University of Wisconsin-Madison, Madison, WI, USA; 11 Department of Surgery, University of Tennessee Graduate School of Medicine, Knoxville, TN, USA; 12 Department of Nursing and Department of Health Sciences, Monmouth University, West Long Branch, NJ, USA

**Keywords:** Global Health, Women, Leadership, Training, Education

## Abstract

**Background:**

Despite advances in gender equality, women still experience inequitable gaps in global health leadership, and barriers to women’s advancement as leaders in global health have been well described in the literature. In 2021, the Johns Hopkins Center for Global Health conducted two virtual working groups for emerging women leaders to share challenges and suggest solutions to advance women’s leadership in global health. In this paper, we present emerging themes from the working groups, provide a framework for the results, and discuss strategies for advancing women’s leadership in global health.

**Objectives:**

The objective of this paper is to synthesize and share the themes of the two working group sessions to provide strategies for improving women’s leadership training and opportunities in the field of global health.

**Methods:**

Approximately 182 women in the global health field participated in two virtual working group sessions hosted by the Johns Hopkins Center for Global Health using the Zoom platform. Participants were divided into virtual breakout rooms and discussed pre-assigned topics related to women’s leadership in global health. The participants then returned to share their ideas in a plenary session. Notes from the breakout rooms and transcripts from the plenary session were analyzed through a participatory and iterative thematic analysis approach.

**Findings:**

We found that the working group participants identified two overarching themes that were critical for emerging women leaders to find success in global health leadership. First, the acquisition of individual essential skills is necessary to advance in their careers. Second, the institutional environments should be setup to encourage and enable women to enter and succeed in leadership roles. The participants also shared suggestions for improving women’s leadership opportunities such as including the use of virtual technologies to increase training and networking opportunities, intersectionality in mentorship and sponsorship, combatting impostor syndrome, and the importance of work-life balance.

**Conclusions:**

Investing in women and their leadership potential has the promise to improve health and wealth at the individual, institutional, and community levels. This manuscript offers lessons and proposes solutions for increasing women’s leadership through improving individual level essential skills and fostering environments in which women leaders can emerge and thrive.

## Background

Despite advocacy, policies, and advances in gender equality over the past decades, women still experience inequitable gaps in the global health profession. The World Health Organization’s “Delivered by Women, Led by Men” report categorizes these gaps that women experience into four strategic areas: leadership, occupation segregation, decent work, and gender pay gap [1,2]. According to the International Labour Organization, careers in health and social services are the fastest growing area for employment opportunities for women around the world [3,4]. In fact, recent data showed that women make up 70% of the global health workforce. However, women remain marginalized in global health leadership, holding only approximately 25% of global health leadership roles [5]. In academic medicine, a field in which publications and self-funding are critical measures for promotion and advancement to leadership roles, women publish high-impact research less often than men, and they are less likely to have first authorships in journal publications [6,7]. Women also receive less funding for research compared to men [8,9]. Even at the highest levels of leadership in non-academic environments, the 2021 Global Health 50/50 report showed that women CEOs in global health organizations face an average annual pay gap of $45,000 compared to their male colleagues, even after controlling for organizations’ revenue in 2021 [10]. These disparities are even more pronounced for women from low- and middle-income countries [11].

Barriers to women’s leadership in different countries and settings have been well established in the literature at the individual and institutional levels [12,13]. Lack of mentorship, workplace power imbalances, work-home responsibility imbalance, and gender bias may occur at each career stage for women and affect career advancement [14,15]. While it is useful to identify the challenges preventing women’s leadership and the need for it, it is even more crucial to create advocacy agendas and implement solutions to enable more women to emerge as successful leaders. Individual, interpersonal, institutional, community, and public policy factors all impact women’s access to and success in leadership roles [5], and a multi-level approach to finding solutions is necessary to see women reach their full potential as leaders.

On the individual level, the advancement of women in global health leadership requires the development of essential skills, historically known as “soft” skills. While hard skills such as technical competencies, expertise, job experiences, and degrees are important, these are required skills needed for anyone to fulfill a leadership position. Women, however, are less often seen as equally competent despite their qualifications, thus limiting their opportunities in leadership spheres [16,17]. Essential skills, on the other hand, are the *“extra”*, non-technical efforts required to attain and succeed in a leadership position such as competencies in personal development, networking, job seeking, management, mentorship, and cultural competency skills. These skills are subjective but reinforce and complement technical competencies. Specific training in these areas is needed. There is also a critical need for institutional support to foster enabling environments for emerging women leaders in the early stages of their careers. The development of enabling environments within which emerging women leaders and trainees may acquire essential skills may provide an important pathway for leadership development [18].

In 2020, the Johns Hopkins Center for Global Health (CGH) started a women in global health leadership program called Emerging Women Leaders in Global Health (EDGE). The EDGE program consists of a seminar series, networking events, a Slack group, and working group opportunities with the goal to establish a network and support women in training and in early stages of their careers from all around the world [18]. In 2021, CGH conducted two virtual working groups to provide space for emerging women leaders to share their challenges and suggest solutions to advance women’s leadership in global health. These sessions were designed to be solutions-oriented, briefly identifying challenges and working towards solutions and strategies for institutions to better support emerging women leaders in the field of global health. In this paper, we present emerging themes from the working groups, provide a framework for the results, and discuss strategies for advancing women leadership in global health.

## Methods

### Working group logistics

The two working group sessions took place on Zoom on January 13, 2021, and March 10, 2021, respectively. The former was an event that was strictly part of the EDGE program while the latter was part of a Consortium of Universities for Global Health (CUGH) Conference Satellite Session and took place immediately after a panel discussion on the topic of women’s leadership in global health. The objective of each of these working group sessions was to identify needs and make recommendations for strategies to improve women’s leadership preparation in the field of global health. Working group members were self-selecting participants who signed up for the events.

During each 40-minute working group session, participants were divided into breakout rooms to discuss the following topics: (1) Strengthening Networks, (2) Building Essential Skills, (3) Authentic/Inclusive Leadership, (4) Building Job-Seeking Skills, (5) Mentorship. The focus areas were chosen based on previous literature that described various barriers to women’s leadership in relevant fields [12,13]. We asked participants to discuss their assigned topics, specifically focusing on how institutions can better support emerging women leaders in global health, and asked one person from each group to take notes. The sub-groups then came together for a plenary session to share their points with the larger group. After each event, we followed up with the note takers via email and asked them to send the notes that they took during the session. We also recorded the plenary discussion on Zoom and downloaded the transcriptions.

### Participants

Eighty-two people from 10 different countries participated in the first working group and self-selected into one of 10 breakout rooms (two rooms were available per topic area to allow for smaller group sizes).

Two hundred twenty-five people attended the CUGH satellite session, and we estimate that 100 participated in the working group and were assigned topic breakout rooms according to the preferences they shared during registration. Data on the countries from which the participants joined was not available for this event.

### Analysis

**Figure 1:**
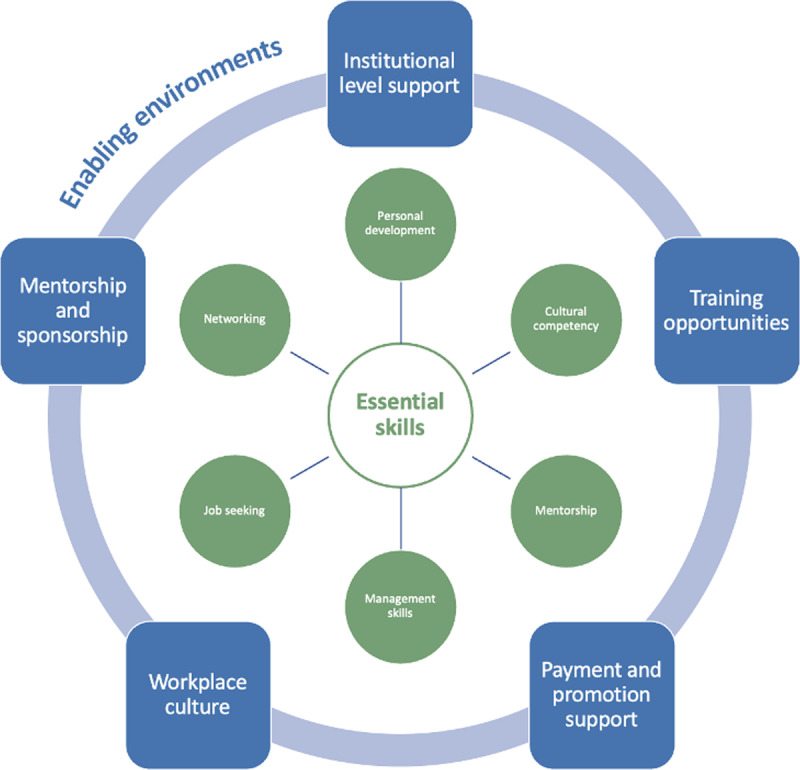
Essential skills and enabling environments to support women’s leadership in global health.

We took an iterative thematic approach to analyzing the notes and transcripts, grouping similar ideas in the data into emergent themes to help understand the experiences, views, and opinions of the working group participants. Upon initial review of the data, the analysis team identified that the working groups’ topics of conversation could be broken into two overarching categories: essential skills needed to become a successful leader and the environments that foster leadership development for women. This became the framework through which we analyzed the emergent sub-themes (Figure 1).

This study was approved by the Institutional Review Board (IRB) at the Johns Hopkins Bloomberg School of Health.

## Results

The working groups identified some key requirements for emerging women leaders to be successful in global health. The requirements fell into two major themes: essential skills needed for advancing as a leader in a global health career and the existence of enabling environments within institutions. The participants also identified solutions and suggestions for helping emerging women leaders to gain these essential skills and for institutions to create enabling environments in their organizations that foster the development of women leaders.

### Essential skills

The working group members identified the following essential skills women need to become a successful leader in the global health field: (1) personal development, (2) networking, (3) job seeking, (4) management, (5) mentorship, and (6) cultural competency.

#### Personal Development Skills

Participants identified personal development, or working on one’s qualities and abilities to help them grow personally and professionally, as essential for emerging leaders in global health careers. Related sub-themes that emerged included the need for self-confidence and self-awareness, communication skills, interpersonal skills, and adaptability.

Some working group participants described having doubts in their abilities or internal beliefs that they are not as competent as others may perceive, which they identified as impostor syndrome. The participants noted that self-confidence is critical for advocating for oneself, stepping into leadership roles, and speaking up in their work environments. They identified the common challenge that women often face where it can be difficult to build the confidence to speak up in meetings or on teams, admitting that they often spent too much time overthinking and missing opportunities to share. The participants shared that self-confidence and self-awareness were also crucial elements for identifying priorities at each stage of their life and career and for learning to set boundaries to establish and maintain work-life balance.

Participants also described the need for communication skills in leadership. These included proficiency in interpersonal communication and public speaking. The ability to communicate scientific findings to different stakeholders and communities was highlighted, and examples of this included communicating to vaccine hesitant groups or vulnerable populations or translating science into policy briefs.

When exploring essential components of interpersonal communication, group members stressed the importance of developing active listening skills as part of good communication to allow others to feel heard when working together. Participants emphasized the importance of learning how to interact with different personalities and work well with others, even when it is challenging. Working group members highlighted that learning how to respectfully disagree was another important element of personal development as a leader. Additionally, the participants identified being able to set boundaries and communicate boundaries as important skills for becoming an effective leader, especially when interacting with more “difficult personalities” by learning when and how to say “no”.

#### Networking Skills

The participants shared that members of the global health community are often altruistic individuals with similar inclinations to make a difference, opening the opportunity for collaboration. Members identified that personal initiative towards networking is needed, and that it is important to get out of their comfort zone to seek out and take advantage of opportunities to network. Participants agreed that networking is a continuous task where one must intentionally cultivate connections, and that it is important to develop and nurture interpersonal relationships in order to experience the benefits of networking. They suggested that emerging women leaders should get to know the culture of their institutions in order to meet potential collaborators, seek out mentors, and engage colleagues who want to work together to lift up others through networking.

One barrier that participants identified to developing effective networks was having feelings of uncertainty related to networking as junior professionals, sharing that networking events often felt siloed and reserved for senior leaders. One approach to overcoming the inhibitions identified among the participants was to find opportunities to present their work at conferences or meetings. The participants also identified social networks such as LinkedIn and Slack for keeping in contact with professional acquaintances after making initial connections. Another suggested way to cultivate relationships was to plan “social hangout times” such as happy hours, dinners, picnics, or other social activities where colleagues can casually interact.

#### Job-seeking dexterity

In the working group discussions, participants indicated the importance of the skills of developing cover letters, résumés, and CVs. They also described the need for interviewing and negotiation skills to successfully land jobs and advance their careers. The common sentiment was that there is the need to highlight and accurately represent the range of skills they possess. For example, participants shared that it was important to know how to present “soft skills’’ such as team building, leadership, and interpersonal skills on their job application documents and during their interviews so that they could effectively communicate the value of those skills to their employers. Participants noted, however, that this was challenging and they wanted opportunities to learn how to best present their varied skills.

The participants also shared challenges with negotiation during the job seeking process. The group highlighted that negotiating was often uncomfortable and that training to learn how to professionally and effectively negotiate salaries and benefits is critical for young professionals to be better compensated for their work.

Some participants suggested that academic programs should put an emphasis on incorporating job-seeking and negotiation skills training into their required course offerings. Others agreed, noting that many academic institutions do have career services available to students but that the services were often generic and lacking contextual relevance to their unique field.

#### Management Skills

The groups discussed the importance of learning both how to manage and how to lead, as well as distinguishing between the two. Practically, they shared that leaders need to learn how to carve out time for completing their own tasks while also managing others. The participants also discussed the importance of decision-making skills in the context of management and leadership. Learning how to make decisions regarding project budgets, personnel challenges and project management, according to the participants, is critical to being an effective leader. In order to build management skills, participants suggested that global health academic programs require management training as part of their course requirements.

#### Individual Mentorship Skills

The participants also shared that learning how to be a good mentor and lifting others up is an essential skill for emerging women leaders. Leaders, according to the group, should take on mentees and empower them to overcome obstacles and improve their skills. Some participants highlighted the importance of lifting other women up to share their ideas, contribute to discussions, and participate in decision making.

*“[In our working group] we talked a lot about lifting up others…and how you can be a leader that creates leadership spaces for other women and how we should do that…in a diverse way, making sure that particularly from a global health perspective, we’re thinking about how we can lift the voices of women in the countries in which we work to surface as the experts and maybe take a step back ourselves.”* (Participant, January 2021 Working Group)

The participants also included that as a leader, it is important to recognize that there will be challenges to fulfill all mentees’ requests and that rather than taking on too much personally and possibly failing, it is better to ensure that their mentees have resources by connecting them to others who can help them on their journeys.

#### Cultural Competency

The ability to understand the culture, values and environment of an institution was emphasized as important for maintaining good cultural competency by the participants. Further, participants shared that it is important to demonstrate cultural competence when working with international colleagues and collaborators, respecting their customs, social norms, and ways of conducting business, among other differences. Another component of cultural competency identified by the participants was the practice of understanding the leadership structures of partner organizations and respecting local leaders, rather than assuming seniority or power. The participants also shared that cultural competency skills for emerging leaders should include the acceptance and celebration that people are different, and the ability to value diversity as a strength.

### Enabling Environments to Support Emerging Women Leaders

Working group participants discussed institutional factors that they believe contribute to an environment that is conducive to promoting women’s leadership. The following themes emerged from the data: (1) Institutional level support, (2) workplace culture, (3) payment and promotion support, (4) a culture of mentorship and sponsorship, (5) training support, and (6) practicing decolonization and intersectionality.

#### Institutional Level Support

According to the participants, one practical factor that helps women to participate fully in the workplace is the implementation of institutional policies that promote inclusion of the gendered experience. Women’s bathrooms and dedicated, comfortable nursing rooms available for lactating people were tangible examples of infrastructure to support women in the built environment. Other inclusive policies that help foster an enabling environment are those that provide support for working parents or caretakers of elderly or sick family members. Participants noted that these responsibilities fall disproportionately on women and that without supportive policies, they worry about falling behind or not being able to fully reach their potential in their careers. Participants mentioned that institutional supports such as student services, specific women’s resources, and provisions to avoid discrimination enhance feelings of inclusion and women’s abilities to successfully advance in their careers.

The participants suggested that by implementing structural support systems, more inclusive behaviors may be fostered, while toxic power and gender dynamics may be avoided. A more formal approach to achieving gender balance was suggested through the development of institutional requirements and/or governmental laws which require gender balance. Navigating power dynamics, according to our data, may also be addressed on both formal and informal levels by encouraging discussions that allow for greater engagement. Promoting discussion at the institutional level between male and female colleagues or between senior and junior colleagues can allow the challenges and concerns faced by specific groups to be shared among colleagues, foster understanding, and stimulate evolution by encouraging individual behavior changes within supportive institutional frameworks.

#### Workplace Culture

Participants identified four core factors that support the development of an enabling workplace culture for women. These include: (1) respectful communication in the workplace, (2) a supportive and affirming environment, (3) a culturally competent environment, and (4) a workplace that values work-life balance.

The participants noted that an enabling workplace culture is one that values respectful communication and clearly communicating expectations. They expressed that while women must build the confidence to speak up, those who are in a position of power should use their position to empower others. They also suggested that condescending attitudes, talking over each other, and dismissing opinions and ideas should be actively discouraged.

Participants indicated that a workplace culture should be supportive and affirming, especially when mistakes are made or help is needed. They shared that, in their experiences, it was easier to admit a mistake when working with a female supervisor. The participants also shared that a workplace culture in which colleagues with privileges (gender, race, age, seniority) support those in less privileged positions is one that helps women to find success in their roles. Women lifting up other women and men supporting women were noted as helpful examples of a supportive workplace culture.

*“One way to support [other women] is to amplify their voices…so when a colleague shares an idea in a meeting or another forum, we can comment positively on their idea or circle back to it if it’s been pushed aside, allowing it to be heard again.”* (Participant, March 2021 Working Group)

They also suggested that creating a sense of community and connecting with people in the workplace through formalized affinity groups and structured discussion and reflection are important strategies for creating a supportive environment. The participants also shared that a culturally competent workplace is key, and that diversity and differences should be valued as strengths. Finally, working group members said that an ideal workplace culture values work-life balance, in policy and in practice.

#### Payment and Promotion Support

Participants noted the challenges they face with inadequate payment and lack of promotion support as barriers to advancing their careers. They suggested that workplaces engage in frequent and transparent discussions about salary pay scales and that they provide adequate compensation for their employees. They indicated that adequate compensation mechanisms should include equity in compensation and career advancement for women in both low- and middle-income countries (LMICs) and high-income countries (HICs), identifying sustainable funding mechanisms for professional advancement, and avoiding the use of stipend pay or volunteer work, which perpetuate norms that ultimately impede women’s progress. Participants also described a desire to see environments in which women are not afraid to ask for appropriate salary compensation.

They also noted the challenges of a lack of clear and comprehensive evaluations and promotion criteria for advancing within an organization. Participants in the academic sphere suggested the need for looking beyond traditional metrics such as authorship and receiving grants. Rather, they would prefer transparent promotion requirements and timelines and an environment in which it is acceptable and normalized to discuss these topics.

#### A Culture of Mentorship and Sponsorship

Mentorship was also highlighted as a core component of an enabling environment that helps emerging women leaders in global health to succeed and thrive in the global health leadership trajectory. While much of participating in mentorship is reliant on how mentors and mentees individually engage and take initiative in the mentorship process, the working group participants focused on the importance of having an environment in which mentorship is expected both formally and informally at the institutional level. Participants mentioned that it can be difficult to initiate and maintain mentorship, particularly outside of formal mentorship structures. While the majority of academic programs have formal structures such as academic advisors and mentors embedded in the system, the support infrastructure weakens when moving on to non-academic settings, making it difficult for early career professionals to identify mentors.

The participants developed solutions to improve mentorship environments. One participant suggested that institutions put a focus on networking during conferences and meetings to help junior colleagues meet potential mentors. Others agreed that deformalizing the mentorship structure would allow for mentors and mentees to have less rigid relationships. Participants noted the importance of having a diversity of mentors for different needs to sustain and enrich their leadership growth rather than relying on just one mentor for everything and suggested more fluid pairings. Some participants proposed that institutions implement “twinning” by pairing students at the same levels from different countries to work together as peer-mentors.

Participants also discussed the distinct differences between mentorship and sponsorship. Mentorship is viewed by the participants as support, guidance, and learning the ropes of a career, while sponsorship is seen as an individual investing in people to provide tangible opportunities to succeed in a career trajectory. While participants felt that mentorship is widely discussed, they identified a need to ensure sponsorship is embedded into systems as well to promote success and growth in the leadership journey. One example of sponsorship that the participants identified was amplifying the voices of others. One tangible example of this was for mentors to bring students or junior colleagues with them to high-level conferences and meetings, and ask them to present their shared work on behalf of, or along with, the mentor. The other participants agreed that gaining skills and experience writing and presenting work in front of high-level colleagues and stakeholders would be an impactful opportunity to make connections and have their voices heard as emerging leaders. Another example shared during the discussion was that mentors could directly connect trainees with professionals in other countries and ask them to provide an opportunity for a job or other connections that their mentee would benefit from. The participants indicated that enabling environments would formalize these systems.

An important caveat that emerged was the need for intersectionality in the mentorship environment. Some participants noted the importance of having either a female mentor or woman of color mentor who may have faced similar challenges or are able to identify with mentees of similar demographics. Other participants suggested that having a mentor from a similar culture may be important in navigating the global health landscape. Participants suggested that if institutions employed higher proportions of diverse senior global health professionals, they would be more available to junior colleagues seeking mentorship from diverse mentors.

#### Training opportunities

Citing the current COVID-19 pandemic, discussions occurred on capitalizing on the global movement to engage online and use technology to create supportive virtual environments that women in global health can use to connect and engage. These online platforms can create and enable ethical and reciprocal training opportunities between women in HICs with their counterparts in LMICs. The participants mentioned the possibility of creating a global training network approach by utilizing online groups on social media platforms such as Facebook, LinkedIn, or Slack channels as a way to foster these kinds of training opportunities.

#### Practicing decolonization and intersectionality

The working group participants noted that in order to approach leadership development in the field of global health equitably, all of the previously suggested solutions should emphasize a decolonization approach. Concerns were raised surrounding tokenistic volunteering and internships, experiences that are used to bolster CVs and perhaps lead to career advancement by those who tout them (usually students and professionals from HICs working in lower income countries). The participants noted the perpetuation of vicious cycles of unequal power dynamics in which roles, jobs and experiences are prioritized to students, volunteers, and visitors from HICs rather than providing opportunities to local talent. The group agreed that when working internationally, existing local leaders should be identified and local structures should be built upon rather than implementing parallel programs. Other solutions included ensuring ethical and reciprocal projects and research opportunities. However, there were still concerns about the complexity of ensuring that unequal power dynamics are not reproduced within those partnerships. Pre-departure and cultural competency training for anyone working in an international context were amongst the targeted solutions discussed. Participants also called for an “intersectional approach,” with a “strengths-based lens” when implementing solutions.

*“Even if it’s hard and there are lots of barriers; I think we should keep bringing up the need for true reciprocity over and over again. I’ve seen it happen only when there is a persistent commitment to it. It involves pretty radical reinvestment of resources, but that is what decolonizing global health is about in my opinion.”* (Participant, March 2021 Working Group)

## Discussion

The need for strong and skilled leadership within global health continues to expand with growing awareness and recognition of the global nature of many health challenges. Women in positions of leadership offer unique perspectives, valuable experience, and renewed innovation. Underrepresentation of women in global health leadership not only affects the production of research publications but also introduces bias to the global health agendas and programming [6]. Women in health leadership positions are more likely to respond directly to community concerns, focus on crucial needs of women, children, and other marginalized groups, allocate funding and attention to research into women’s health issues, and incorporate a focus on important public health outreach such as health and nutrition education [19]. Most recently, evidence has shown that countries with women leaders have fared better facing the COVID-19 pandemic [20]. Gender equity in global health leadership is therefore essential to adequately addressing global health issues and those disparities that impact female populations across the globe. Both women and global health settings are diverse, requiring a well-considered and intersectional approach to empowering women leaders in global health at both individual and institutional levels. Structural level factors that create enabling environments must be developed to allow women to develop these individual-level skills and emerge as leaders just as much as men.

###  

#### Essential Skills

The two working group sessions analyzed here revealed key insights into the essential skills that the group felt are foundational for advancing as global health professionals. While leadership skills have been previously surveyed among academic and community health settings, solutions to successfully foster women’s essential skills have been under-reported [21]. Our analysis shows that participants value developing several overarching areas of individual-level essential skills that are needed for advancing as a leader in a global health career such as personal development skills, job-seeking dexterity, communication abilities, the ability to successfully network, and an understanding of cultural competency.

#### Putting barriers to the individual into an institutional context

A number of challenges to women’s leadership were described by the working group participants. It is important to note that the barriers that we mention above exist within the context of larger, systemic barriers. Most barriers cannot be solved without systemic changes; the burden of rising to leadership, thus, is not on women individually or as a group. Ecological, societal, and organizational changes are needed to ensure individuals have opportunities to successfully develop their strengths as leaders and optimize individual capabilities. Meta-level solutions are essential to address the many institutional, community, and public policy factors that create barriers against women’s advancement in leadership positions in global health.

Deep-rooted institutional structures often contribute to power imbalances and disparities in leadership opportunities within the health sector [22]. Though evidence suggests otherwise, societal definitions of the qualities found in a good leader are often skewed towards a more traditionally masculine approach [23,24]. On the other hand, gender transformative leadership addresses social and cultural norms, bias, and deep-rooted inequalities with the goal of advancing gender equality and women’s rights [25]. In the context of global health, this may involve challenging imbalances in power to address gender disparities and creating opportunities for women to strengthen their confidence, skills, and access to tools needed to succeed and advocate for themselves, key areas identified by working group participants. Expanding the definition of a leader at the meta-level, advocating for greater awareness, and addressing bias and system-wide barriers can allow women to define and succeed at leadership on terms that are meaningful to them.

Workplaces should also be structured so that women feel included, safe, respected, and valued to empower them to have a voice in directing their work. Equity of rights, benefits, opportunities, and obligations is critical for encouraging different approaches and creating supportive, productive environments where one can optimize their skills [26].

#### Solution: Use of technology for women-inclusive training and networking opportunities

The importance of availability and access to training and networking opportunities was highlighted by the members of the working groups. The WHO maintains that to enable women to achieve leadership in global health, there is a need to develop formal and informal networks for women’s leadership development [5]. The working group participants suggested that institutions should support and enable opportunities for methods of networking. One of the prominent solutions suggested by the working group participants was taking advantage of internet technology to include more women in leadership training and networking opportunities.

This apparent affinity for virtual opportunities may have been because the working groups were conducted virtually and supported by a virtual leadership program that was created out of a desire to connect while socially distancing during the COVID-19 pandemic, or perhaps due to the gap in access that women face as it relates to such opportunities. It has been previously established that women find it harder to access training for career advancement due to reasons such as cost, timing, location, conflicts with other responsibilities outside work, and gender-bias in sending staff for training [1]. The unprecedented use of internet technology for communication in the wake of the COVID-19 pandemic has the potential to strengthen leadership preparation for women, especially in the field of global health, providing distance training and networking that would not have been possible if travel was required [27] However, while online meeting platforms such as Zoom have been helpful to connect people in more inclusive ways, “Zoom fatigue” can be a challenge and there is evidence to suggest that this phenomenon is felt more severely by women compared to men [28].

This is well articulated in the development and use of platforms such as the Women in Global Health Slack network, which is an initiative that has the capacity to provide information on opportunities for growth such as mentorship, networking events, and career opportunities that are key to progression in both academic and non-academic careers. In addition, the Women’s Global Health Leadership seminars provide invaluable opportunities for women to learn from each other, particularly from more experienced women leaders as well as from peers. These two opportunities have the potential to provide women a platform to create alliances with other women for support and catalyze organizational change [5].

#### Solution: Intersectionality in mentorship and sponsorship

While mentorship is an integral component in providing guidance and support to early career women, sponsorship can be career-defining and monumental [29,30]. Specifically, sponsorship refers to having an advocate/sponsor that is actively recommending high visibility, career advancement opportunities including leadership involvement, invitation panels, and awards [30,31]. The findings of this study show that there is a need to amplify voices and intentionally bring hyper-visibility for female students and junior colleagues. This is critical in promoting confidence among young individuals who are doing the work and getting rewarded as a result of the opportunity.

Additionally, men can play a critical role in ensuring that women leaders’ voices are heard and represented. Studies have indicated that men are more likely to be sponsored by senior colleagues compared to women [32]. As suggested by the working group participants in this study, men should openly advocate opportunities for their female counterparts. One way to be a better advocate is to refuse to participate in conferences or other venues that do not intentionally include diverse colleagues or offer the opportunity to their female colleagues who are often under-represented on platforms such as panels, speaking agendas, or high-level meetings. Promoting active collaboration with men and senior colleagues is a pathway to improving the leadership career trajectory for women in global health. Structural changes in the workplace to incentivize mentorship and sponsorship are also recognized ways to elevate women leaders [14].

There are other power dynamics that exist among women themselves in which an intersectional approach is important to elevating women of diverse race, religion, age, ability, gender identity, and sexual orientation [33]. Cisgender white women should also play critical roles to elevate their non-cisgender, non-white counterparts.

#### Solution: Combating imposter syndrome

Some working group participants described having doubts in their abilities. This struggle with self-silencing or a lack of self-belief in one’s abilities can contribute to “Imposter Syndrome” where one feels she is not deserving of the power, credibility, or authority to lead [19]. To combat these internal doubts in one’s ability, participants stressed that emerging global health leaders need to be able to identify what they need to succeed and gain the confidence and skills to advocate for the things they require to support their success. On an individual level, coaching and mentoring can help women increase their confidence and believe in themselves and their abilities. Peer networks, professional associations, and leadership trainings that take women’s unique strengths and perspectives in mind can also boost women’s confidence and preparation for advancing their careers as leaders in global health.

#### Solution: Work-life balance and family-forward policies

The working group participants believe that women generally carry a greater burden of household and family responsibilities and frequently spend disproportionate amounts of time doing unpaid care work in addition to their formal employment, leading to difficulty with work-life balance. This is a sentiment consistent with the literature [27,34,35]. Work-life balance and successful integration of one’s life priorities is crucial for an individual’s well-being and livelihood as well as having broader impacts on gender equity, health systems, and the delivery of quality care [1].

On a systemic level, steps that foster an organizational culture that promotes norms that support all employees in successfully integrating their careers with responsibilities outside of the workplace can provide some of the foundations for building a career that allows for time management in a personally meaningful way, one of the key themes highlighted by working group participants. Family-friendly policies that promote a flexible work environment and structural solutions such as childcare and funding for parental leave as well as addressing institutional barriers such as wage gaps can create conditions that are more conducive to balancing work and home life [36]. There must be a workplace culture of respect, support, and valuing for women who take a “non-traditional” career path in global health to balance other responsibilities in their life. This may involve adjusting the culture of metrics for advancement, normalizing flexible work hours and telecommuting, and incorporating equity into policies at all levels.

## Conclusion

Empowering women, especially early-career professionals, to obtain and succeed in global health leadership positions is a key component of effectively addressing the health of the community at large. Women are clearly essential to the functioning of families, communities, and health care systems. Their expertise, lived experiences, and insights are invaluable, but they are inequitably represented among leadership positions.

Overcoming the barriers that limit women leaders in global health requires solutions that address the larger environment to empower a foundation for the individual to build and utilize their training and essential skills. True sustainable change can only be maintained by assessing current norms, addressing existing inequities, and creating more inclusive and supportive cultures and systems at organizational and societal levels. This includes examining internal systems and processes and adapting them to create the most enabling environments for women to reach their full leadership abilities. Taking an intersectional approach working within the larger ecosystem to advance gender equality allows opportunities to surface to help tackle the barriers women face in the global health workforce while also addressing global health challenges.

## References

[1] World Health Organization. Delivered by Women, Led by Men: A Gender and Equity Analysis of the Global Health and Social Workforce. World Health Organization; 2019. Accessed December 11, 2021. https://apps.who.int/iris/bitstream/handle/10665/311322/9789241515467-eng.pdf.

[2] Boniol M, McIsaac M, Xu L, Wuliji T, Diallo K, Campbell J. Gender equity in the health workforce: Analysis of 104 countries. Published online 2019.

[3] International Labour Office. Improving Employment and Working Conditions in Health Services: Report for Discussion at the Tripartite Meeting on Improving Employment and Working Conditions in Health Services. International Labour Organization; 2017. Accessed December 9, 2021. https://www.ilo.org/wcmsp5/groups/public/---ed_dialogue/---sector/documents/publication/wcms_548288.pdf.

[4] International Labour Office. World Employment and Social Outlook: Trends 2020. World Employ Soc Outlook. 2020; 2020(1): 1–10. DOI: 10.1002/wow3.147

[5] World Health Organization. Closing the leadership gap: Gender equity and leadership in the global health and care workforce: policy action paper, June 2021. Published online June 2021. Accessed December 10, 2021. https://www.who.int/publications/i/item/9789240025905.

[6] Penny M, Jeffries R, Grant J, Davies SC. Women and academic medicine: A review of the evidence on female representation. J R Soc Med. 2014; 107(7): 259–263. DOI: 10.1177/0141076814528893 24739380 PMC4093756

[7] Filardo G, da Graca B, Sass DM, Pollock BD, Smith EB, Martinez MA-M. Trends and comparison of female first authorship in high impact medical journals: Observational study (1994–2014). BMJ. 2016; 352: i847. DOI: 10.1136/bmj.i847 26935100 PMC4775869

[8] Kaatz A, Gutierrez B, Carnes M. Threats to objectivity in peer review: The case of gender. Trends Pharmacol Sci. 2014; 35(8): 371–373. DOI: 10.1016/j.tips.2014.06.005 25086743 PMC4552397

[9] Morgan R, Hawkins K, Lundine J. The foundation and consequences of gender bias in grant peer review processes. CMAJ. 2018; 190(16): E487–E488. DOI: 10.1503/cmaj.180188 29685908 PMC5915245

[10] Global Health 50/50. Gender Equality: Flying Blind in a Time of Crisis, The Global Health 50/50 Report 2021. University College London Centre for Gender and Global Health.; 2021. DOI: 10.2499/p15738coll2.134569

[11] Shumba CS, Lusambili AM. Not enough traction: Barriers that aspiring researchers from low- and middle-income countries face in global health research. Journal of Global Health Economics and Policy. Published online July 26, 2021. DOI: 10.52872/001c.25802

[12] Maheshwari G. A review of literature on women’s leadership in higher education in developed countries and in Vietnam: Barriers and enablers. Educational Management Administration & Leadership. Published online June 8. DOI: 10.1177/17411432211021418

[13] Mate SE, McDonald M, Do T. The barriers and enablers to career and leadership development. Int J of Org Analysis. 2019; 27(4): 857–874. DOI: 10.1108/IJOA-07-2018-1475

[14] Moyer CA, Abedini NC, Youngblood J, et al. Advancing women leaders in global health: getting to solutions. Ann Glob Health. 2018; 84(4): 743–752. DOI: 10.29024/aogh.2384 30779525 PMC6748298

[15] Lundine J, Bourgeault IL, Clark J, Heidari S, Balabanova D. The gendered system of academic publishing. Lancet. 2018; 391(10132): 1754–1756. DOI: 10.1016/S0140-6736(18)30950-4 29739551

[16] Quadlin N. The mark of a woman’s record: gender and academic performance in hiring. Am Sociol Rev. 2018; 83(2). DOI: 10.1177/0003122418762291

[17] Cardel MI, Dhurandhar E, Yarar-Fisher C, et al. Turning Chutes into Ladders for Women Faculty: A Review and Roadmap for Equity in Academia. J Womens Health (Larchmt). 2020; 29(5): 721–733. DOI: 10.1089/jwh.2019.8027 32043918 PMC7247039

[18] Kalbarczyk A, Harrison M, Chung E, et al. Supporting Women’s Leadership Development in Global Health through Virtual Events and Near-Peer Networking. Ann Glob Health. 2022; 88(1). DOI: 10.5334/aogh.3397 PMC8740638 35083128

[19] Batson A, Gupta GR, Barry M. More women must lead in global health: A focus on strategies to empower women leaders and advance gender equality. Ann Glob Health. 2021; 87(1): 67. DOI: 10.5334/aogh.3213 34307070 PMC8284502

[20] Purkayastha S, Salvatore M, Mukherjee B. Are women leaders significantly better at controlling the contagion during the COVID-19 pandemic? J Health Soc Sci. 2020; 5(2): 231–240. DOI: 10.1101/2020.06.06.20124487 32875269 PMC7457824

[21] Johnson A. Scoping Review: Women’s Leadership Training in Global Health. Annals of Global Health. Published online 2022.

[22] Newman C, Chama PK, Mugisha M, Matsiko CW, Oketcho V. Reasons behind current gender imbalances in senior global health roles and the practice and policy changes that can catalyze organizational change. Glob Health Epidemiol. 2017; 2: e19. DOI: 10.1017/gheg.2017.11 PMC5870424 29868225

[23] Appelbaum SH, Audet L, Miller JC. Gender and leadership? Leadership and gender? A journey through the landscape of theories. Leadership & Org Development J. 2003; 24(1): 43–51. DOI: 10.1108/01437730310457320

[24] Verma SP, Krishnan VR. Transformational leadership and follower’s organizational commitment: Role of leader’s gender. NMIMS Management Review. 2013; 23: 91–112.

[25] Wakefield S. Transformative and feminist leadership for women’s rights. Oxfam America Research Backgrounder Series. Published online 2017.

[26] Date-Bah E, Zhang J, Heide I. ABC of Women Workers’ Rights and Gender Equality. International Labour Organization; 2000.

[27] Downs JA, Reif LK, Hokororo A, Fitzgerald DW. Increasing women in leadership in global health. Acad Med. 2014; 89(8): 1103–1107. DOI: 10.1097/ACM.0000000000000369 24918761 PMC4167801

[28] Fauville G, Luo M, Queiroz ACM, Bailenson JN, Hancock J. Nonverbal Mechanisms Predict Zoom Fatigue and Explain Why Women Experience Higher Levels than Men. SSRN Journal. Published online 2021. DOI: 10.2139/ssrn.3820035

[29] Perry RE, Parikh JR. Sponsorship: A proven strategy for promoting career advancement and diversity in radiology. J Am Coll Radiol. 2019; 16(8): 1102–1107. DOI: 10.1016/j.jacr.2019.04.018 31092339

[30] Ang J. Why career sponsorship matters for advancing women. Women and Business. 2018; 1(4): 36–43.

[31] Ayyala MS, Skarupski K, Bodurtha JN, et al. Mentorship is not enough: exploring sponsorship and its role in career advancement in academic medicine. Acad Med. 2019; 94(1): 94–100. DOI: 10.1097/ACM.0000000000002398 30095456

[32] Ang J. Career sponsorship: an effective way for developing women leaders. In: Sendjaya S, (ed.), Leading for High Performance in Asia: Contemporary Research and Evidence-Based Practices. Singapore: Springer; 2019: 89–105. DOI: 10.1007/978-981-13-6074-9_5

[33] Benschop Y. Grand challenges, feminist answers. Organization Theory. 2021; 2(3): 263178772110203. DOI: 10.1177/26317877211020323

[34] Bianchi SM, Sayer LC, Milkie MA, Robinson JP. Housework: who did, does or will do it, and how much does it matter? Soc Forces. 2012; 91(1): 55–63. DOI: 10.1093/sf/sos120 25429165 PMC4242525

[35] Schiebinger L, Gilmartin SK. Housework is an academic issue. Academe. 2010; 96(1): 39–44.

[36] Carr PL, Gunn C, Raj A, Kaplan S, Freund KM. Recruitment, promotion, and retention of women in academic medicine: How institutions are addressing gender disparities. Women’s Health Issues. 2017; 27(3): 374–381. DOI: 10.1016/j.whi.2016.11.003 28063849 PMC5435548

